# Genetic Polymorphisms in Vitamin D Metabolism and Signaling Genes and Risk of Breast Cancer: A Nested Case-Control Study

**DOI:** 10.1371/journal.pone.0140478

**Published:** 2015-10-21

**Authors:** Tess V. Clendenen, Wenzhen Ge, Karen L. Koenig, Tomas Axelsson, Mengling Liu, Yelena Afanasyeva, Anne Andersson, Alan A. Arslan, Yu Chen, Göran Hallmans, Per Lenner, Tomas Kirchhoff, Eva Lundin, Roy E. Shore, Malin Sund, Anne Zeleniuch-Jacquotte

**Affiliations:** 1 Department of Population Health, New York University School of Medicine, New York, New York, United States of America; 2 New York University Cancer Institute, New York University School of Medicine, New York, New York, United States of America; 3 Department of Molecular Medicine, Science for Life Laboratory, Uppsala University, Uppsala, Sweden; 4 Department of Oncology, Umeå University Hospital, Umeå, Sweden; 5 Department of Obstetrics and Gynecology, New York University School of Medicine, New York, New York, United States of America; 6 Department of Public Health and Clinical Medicine/Nutritional Research, Umeå University, Umeå, Sweden; 7 Department of Medical Biosciences/Pathology, Umeå University, Umeå, Sweden; 8 Radiation Effects Research Foundation, Minami-ku, Hiroshima, Japan; 9 Department of Surgery, Umeå University Hospital, Umeå, Sweden; University of Arkansas for Medical Sciences, UNITED STATES

## Abstract

Genetic polymorphisms in vitamin D metabolism and signaling genes have been inconsistently associated with risk of breast cancer, though few studies have examined SNPs in vitamin D-related genes other than the vitamin D receptor (VDR) gene and particularly have not examined the association with the retinoid X receptor alpha (RXRA) gene which may be a key vitamin D pathway gene. We conducted a nested case-control study of 734 cases and 1435 individually matched controls from a population-based prospective cohort study, the Northern Sweden Mammary Screening Cohort. Tag and functional SNPs were genotyped for the VDR, cytochrome p450 24A1 (CYP24A1), and RXRA genes. We also genotyped specific SNPs in four other genes related to vitamin D metabolism and signaling (GC/VDBP, CYP2R1, DHCR7, and CYP27B1). SNPs in the CYP2R1, DHCR7, and VDBP gene regions that were associated with circulating 25(OH)D concentration in GWAS were also associated with plasma 25(OH)D in our study (p-trend <0.005). After taking into account the false discovery rate, these SNPs were not significantly associated with breast cancer risk, nor were any of the other SNPs or haplotypes in VDR, RXRA, and CYP24A1. We observed no statistically significant associations between polymorphisms or haplotypes in key vitamin D-related genes and risk of breast cancer. These results, combined with the observation in this cohort and most other prospective studies of no association of circulating 25(OH)D with breast cancer risk, do not support an association between vitamin D and breast cancer risk.

## Introduction

**Fig 1:**
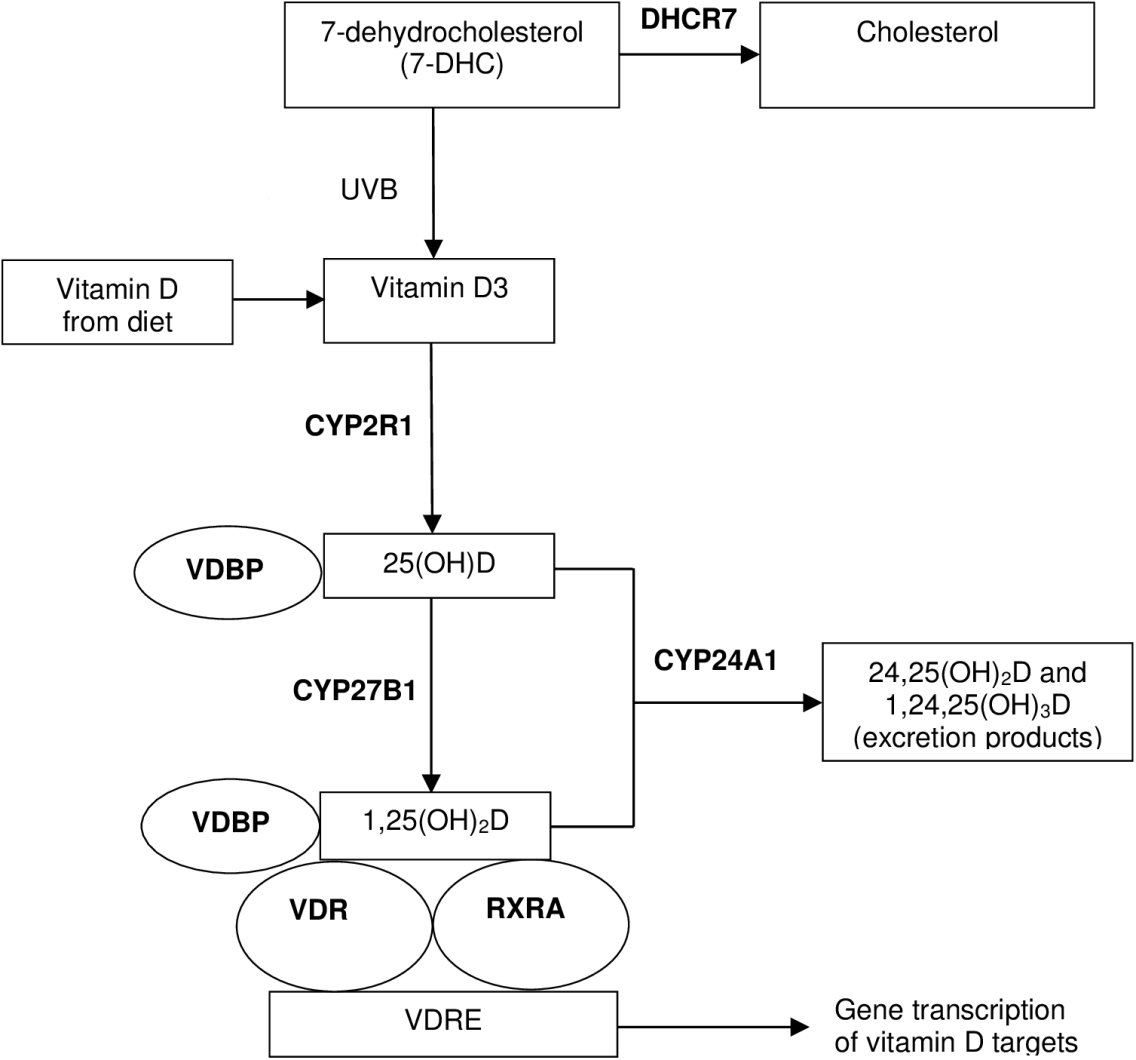
Vitamin D pathway. Vitamin D_3_ (cholecalciferol) is produced in the skin from UV photoconversion of 7-dehydrocholesterol (7-DHC), which can be converted to cholesterol through a process mediated by 7-dehydrochlesterol reductase (DHCR7), or obtained through diet. Vitamin D_3_, as well as vitamin D_2_ from plant sources and dietary supplements, is metabolized in two hydroxylation steps. The first hydroxylation occurs in the liver largely by CYP2R1-mediated 25-hydroxylase action to form the most abundant circulating metabolite, 25(OH)D [7] and the second in the kidney, breast, and other tissues by CYP27B1 to form the active metabolite, 1,25(OH)_2_D [8, 9]. The molecular actions of 1,25(OH)_2_D occur upon its binding to the vitamin D receptor (VDR), prompting the association of the VDR with the retinoid X receptor alpha (RXRA), to activate or suppress transcription of downstream gene targets. 25(OH)D and 1,25(OH)_2_D are broken down into 24-hydroxylated products by CYP24A1 and targeted for excretion [10]. Vitamin D and its metabolites are bound with varying affinities and transported in the circulation by the vitamin D-binding protein (VDBP) [11–13].

*In vitro* and *in vivo* studies suggest that vitamin D may have a protective effect against breast cancer due to its role in regulating cell growth and apoptosis [1]. However, most prospective epidemiological studies, including our own [2], that measured prediagnostic circulating 25(OH)D concentration, the best indicator of vitamin D status, did not observe any association with breast cancer risk [2–6]. Single nucleotide polymorphisms (SNPs) in genes encoding proteins that act downstream of 25(OH)D production, including proteins related to vitamin D transport (*e*.*g*., vitamin D binding protein, GC/VDBP), metabolism (*e*.*g*., 24-hydroxylase, CYP24A1), and signaling (*e*.*g*., vitamin D receptor, VDR, and retinoid X receptor alpha, RXRA) may affect the inter-individual biological effects of vitamin D and thus influence risk (Fig 1, [7–13]). The associations between most candidate SNPs in vitamin D-related genes and risk of breast cancer have been null or mixed in previous studies [14–19]. Few studies, though, have examined risk in relation to SNPs in vitamin D-related genes other than VDR [18, 20–25], and, to our knowledge, none have looked at RXRA SNPs.

The purpose of this study was to examine the relationship between polymorphic variation in several vitamin D-related genes and risk of breast cancer in a case-control study nested within the Northern Sweden Mammary Screening Cohort, one of the two cohorts included in our previously published analysis of circulating 25(OH)D and breast cancer risk [2]. We performed an analysis of tag SNPs and potential functional SNPs as well as haplotypes in three genes that act downstream of 25(OH)D formation (VDR, CYP24A1, and RXRA) and of the only SNP in the CYP27B1 gene with common variation. We also genotyped the candidate SNPs in gene regions that were related to circulating 25(OH)D concentrations in genome wide association studies (GWAS) and/or other epidemiological studies (*i*.*e*., a 25-hydroxylase [CYP2R1], GC/VDBP, and 7-dehydrocholesterol reductase [DHCR7] [26–31]). We also evaluated whether the association between these genetic polymorphisms and breast cancer risk varied according to the menopausal status of the subjects or the estrogen receptor status of the breast tumors.

## Methods

### Study subjects

The Northern Sweden Mammary Screening Cohort (NSMSC) is a population-based prospective cohort in Västerbotten County in Northern Sweden, and has been described in detail [2]. Breast cancer cases were identified through linkage with the Swedish Cancer Registry. As of January 2010, there were 736 cases of invasive breast cancer (n = 734 with DNA). Incidence density sampling was used to select two controls for each case from the appropriate risk set, i.e. members of the NSMSC who were alive and free cancer at the time of the case’s diagnosis and who matched the case on age at enrollment (±6 months), date of enrollment/first blood donation (± 1 month), and number (1, 2+) and dates of subsequent blood donations. Though menopausal status was not a matching factor in early breast cancer studies in this cohort, at least one control matched the case on menopausal status (pre/peri- vs. post-menopausal) at blood donation for 94% of the matched sets. In total, 734 cases and 1435 controls were included in this study. The Regional Ethics Committee of the University of Umeå, Sweden, and the Swedish Data Inspection Board approved this study. All participants provided written informed consent.

### Selection of genetic variants and genotyping

We selected two sets of vitamin D-related genes: 1) Those that were found in GWAS or other large scale epidemiological studies to be associated with 25(OH)D concentrations. For these genes, we genotyped the individual SNPs that had been associated with 25(OH)D in previous studies and evaluated the association of these SNPs both with 25(OH)D concentration and with breast cancer risk in our study group. 2) Genes that act downstream of 25(OH)D, and therefore may reflect vitamin D effects that are not captured in studies that measure 25(OH)D concentrations. For these genes, we examined the association of tag and functional SNPs with breast cancer risk. Specifically we selected the VDR and its co-receptor, RXRA, the vitamin D breakdown enzyme CYP24A1, and CYP27B1, which is involved in the hydroxylation of 25(OH)D to its active metabolite Other genes associated with the vitamin D pathway that may act downstream of 25(OH)D formation are not necessarily specific to vitamin D actions (e.g. inflammation mediators) and thus were not genotyped in our study of vitamin D-related genes. Tag SNPs were selected for VDR, CYP24A1, and RXRA genes plus the regions 10kb upstream and 10kb downstream using the software program tagger (r^2^ threshold of 0.8) and using the Hapmap CEU population (1000 Genomes data was not available at the time of tag SNP selection for this study). We also selected potentially functional SNPs in these genes identified through literature searches and *in silico* databases (dbSNP, last accessed in 2014, F-SNP http://compbio.cs.queensu.ca/F-SNP/, last accessed 6/2008, and PupaSuite http://pupasuite.bioinfo.cipf.es/, last accessed 6/2009). SNPs with minor allele frequencies (MAF) <5% in the Hapmap CEU population were excluded. For CYP27B1, we genotyped the only SNP in the gene with MAF >5%. We did not include ancestry-specific SNPs because the vast majority of women in this study were from the same region in Northern Sweden.

DNA was isolated from buffy coat material at the University of Umeå. Genotyping was performed at Uppsala University Hospital SNP&SEQ Technology Platform using the Illumina Golden Gate assay and GenomeStudio 2011.1 software [32].

**Fig 2:**
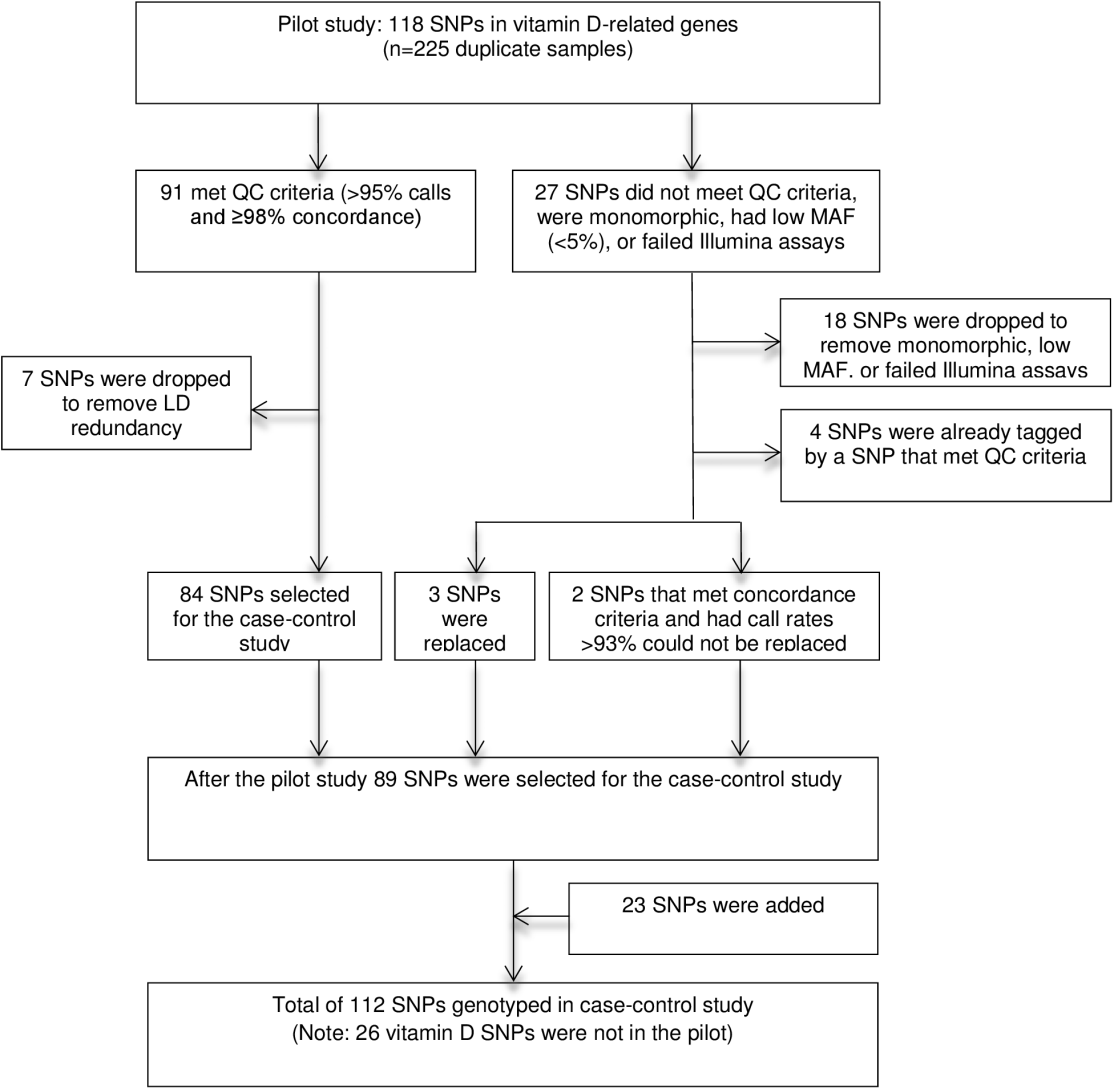
SNPs genotyped in pilot and case-control studies.

Prior to analyzing case-control samples, a pilot study was conducted to assess genotyping accuracy and reproducibility of the selected SNPs using the Golden Gate assay and NSMSC samples. For the pilot study, DNA samples from 225 women were genotyped in duplicate (450 samples total) for 118 SNPs. The samples were labeled to conceal the identity of the duplicates from laboratory personnel. SNPs that met *a priori* quality control criteria (call rate ≥95% and concordance >98%) were included in the case-control study and those that did not were replaced unless they were already tagged by other genetic variants (Fig 2).

For the case-control study, a total of 112 SNPs were genotyped. This incuded 6 candidate SNPs in 3 gene regions (3 in GC/VDBP, 1 in CYP2R1, and 2 in DHCR7) which had been associated with 25(OH)D concentrations in GWAS [26, 27]; and 106 tag or functional SNPs in genes that act downstream of 25(OH)D: 42 in VDR, 31 in CYP24A1, 32 in RXRA, and 1 in CYP27B1. Genotype call rates in the case-control study were between 95–100%. Concordance for 148 blinded duplicate samples (296 samples total) was 100% for all but three SNPs (≥98.5%). Genotype frequencies in controls were consistent with Hardy-Weinberg equilibrium (p>0.00045 (0.05/112 SNPs)).

### Statistical analysis

#### SNPs and 25(OH)D concentration

Season-adjusted geometric means and 95% confidence intervals (CIs) of circulating 25(OH)D [2] were calculated according to genotype in the controls.

#### SNPs and breast cancer risk

Conditional logistic regression was used to calculate odds ratios (OR) for breast cancer. Two indicator variables were used to calculate ORs by genotype, with no assumption of genetic model. For SNPs with the homozygous variant genotype occurring for ≤10 women, the heterozygous and homozygous variant genotypes were combined. Tests for trend assumed an additive model (*i*.*e*., 0, 1, or 2 copies of the variant allele, 1 degree of freedom). Subjects with missing genotype data for any SNP (<5%) were excluded. Multiple imputation, using fully conditional specification [33], was used to impute missing data for the following covariates in the multivariate-adjusted model: hormone replacement therapy (HRT) use (ever/never), age at menarche (continuous), parity/age at first full-term pregnancy (categorical as shown in Table 1), and body mass index (BMI, continuous log_2_ scale) (3–6% missing data). We compared models with imputed data for covariates vs. complete case analyses and did not observe any appreciable difference in the odds ratios. Thus, we show the results with imputed covariate data for multivariate models which allowed us to include all of the women in the analysis. Other variables considered as potential confounders (shown in Table 1) did not appreciably affect the odds ratios and were not included in final models. Categorization for continuous variables in Table 1 was done using tertiles, except for BMI, for which we used standard clinical categories: normal weight (<25 kg/m2), overweight (25–30 kg/m2), and obese (>30 kg/m2); and for age at sampling, diagnosis, and first full term pregnancy, for which we used 5–10 year intervals. The false discovery rate (FDR) approach was used to account for multiple testing [34].

**Table 1:** Description of case and control subjects, NSMSC.

Characteristics ^a^	Cases (n = 734)	Controls (n = 1435)	p value ^b^
	N (%)	N (%)	
Age at blood donation, years			Matched
< 55	315 (42.9%)	594 (41.4%)	
55–60	182 (24.8%)	365 (25.4%)	
> 60	237 (32.3%)	476 (33.2%)	
Age at diagnosis, years			
< 55	115 (15.7%)		
55–59	136 (18.5%)		
60–64	176 (24.0%)		
> 65	307 (41.8%)		
Height, cm			0.07
< 162	217 (30.1%)	489 (34.9%)	
162–167	300 (41.6%)	568 (40.5%)	
> 167	204 (28.3%)	345 (24.6%)	
Body mass index, kg/m^2^			0.85
Pre- or peri-menopausal at enrollment			
< 25	93 (53.4%)	162 (51.4%)	
25–30	65 (37.4%)	116 (36.8%)	
> 30	16 (9.2%)	37 (11.8%)	
Postmenopausal at enrollment			0.08
< 25	245 (45.9%)	535 (50.0%)	
25–30	206 (38.6%)	413 (38.6%)	
> 30	83 (15.5%)	122 (11.4%)	
Age at menarche, years			< 0.01
< 13	234 (32.8%)	369 (26.6%)	
13	191 (26.8%)	359 (25.9%)	
> 13	288 (40.4%)	660 (47.6%)	
Menopausal status at enrollment			Matched ^e^
Pre- and peri-menopausal	181 (24.7%)	320 (22.3%)	
Postmenopausal	553 (75.3%)	1115 (77.7%)	
Age at menopause, years			0.36
≤ 49	213 (41.0%)	406 (40.3%)	
49–51	108 (20.8%)	244 (24.2%)	
≥ 51	198 (38.2%)	358 (35.5%)	
Parity			0.07
Nulliparous	55 (8.1%)	83 (6.1%)	
Parous	628 (91.9%)	1285 (93.9%)	
Age at first full term pregnancy, years ^c^			0.11
≤ 20	88 (14.7%)	242 (19.6%)	
21–25	300 (50.1%)	551 (44.7%)	
26–30	147 (24.5%)	335 (27.1%)	
> 30	64 (10.7%)	106 (8.6%)	
Alcohol intake, g/day			0.17
< 1	212 (47.9%)	402 (44.5%)	
1–3	96 (21.7%)	200 (22.2%)	
> 3	135 (30.5%)	301 (33.3%)	
Past oral contraceptive use			0.81
Ever	382 (53.4%)	737 (52.2%)	
Never	333 (46.6%)	674 (47.8%)	
Hormone replacement therapy use ^d^			< 0.001
Ever	272 (39.6%)	402 (29.4%)	
Never	415 (60.4%)	964 (70.6%)	
Estrogen receptor (ER) status			
Positive	503 (78.8%)		
Negative	135 (21.2%)		
Plasma season-adjusted 25(OH)D level, nmol/L			0.44
< 46	222 (32.2%)	420 (32.5%)	
46–60	248 (35.9%)	420 (32.5%)	
> 60	220 (31.9%)	452 (35.0%)	

a. Missing values: < 5% for height, BMI, age at menarche, age at first full term pregnancy, and OC use; <10% for plasma 25(OH)D level and HRT use; 30% for age at menopause, 38% for alcohol intake, and 13% for ER status

b. P value for trend was calculated by conditional logistic regression using ordered categories; two-sided

c. Among ever parous women

d. Among postmenopausal women

e. Menopausal status is partially matched. Cases identified/selected more recently have two controls/case matched on menopausal status

#### Haplotypes and breast cancer risk

Data from the 1000 Genomes Project (CEU population, October 2012 release) was used as the reference panel to define haplotype block boundaries for the genes for which multiple tag SNPs were genotyped (VDR, RXRA, and CYP24A1). Linkage disequilibrium (LD) patterns and pairwise r^2^ values were similar for our study controls and the 1000 Genomes subjects (see S1 Fig). Blocks for which we genotyped at least three SNPs were included in our analysis of haplotypes and risk. Haplotype frequencies for cases and controls as well as the associations between haplotypes and risk were estimated using HaploStats 1.6.8 in R 3.0.2, with the most common haplotype in each block as the reference. Because conditional logistic regression analysis is not accommodated in this software, and because we did not observe any appreciable difference in ORs for individual SNPs using conditional vs. unconditional logistic regression (adjusting for the matching factors), ORs and 95% CIs were calculated using unconditional logistic regression models. These analyses adjusted for the matching factors (age and menopausal status at blood donation), as well as for other breast cancer risk factors.

## Results

Table 1 shows selected case and control subjects’ characteristics. Consistent with expectation, a significantly higher proportion of cases than controls had a history of hormone replacement therapy use (40% vs. 29%, p<0.0001) and cases had, on average, an earlier age at menarche than controls (p_trend_<0.01). Among postmenopausal women, cases tended to have a higher BMI than controls (p = 0.08). The proportion of nulliparous women was higher in cases than controls (8% vs. 6%, p = 0.07), and among parous women, cases tended to have been older at first full term pregnancy than controls (p_trend_ = 0.11). No significant difference was observed between cases and controls for age at menopause, alcohol intake, past oral contraceptive use, or plasma 25(OH)D.

SNPs associated with 25(OH)D concentration in GWAS [26, 27] were also significantly associated with 25(OH)D concentration in our study, except CYP24A1 rs6013897 (Table 2). Adjustment for age and BMI did not change the p-values appreciably (data not shown). No significant trends in breast cancer risk were observed across genotypes for any of these SNPs after FDR adjustment (S1 Table).

**Table 2:** Season-adjusted 25(OH)D geometric means by genotype (for SNPs associated with 25(OH)D in GWAS) among controls (n = 1292).

Gene/SNP	Genotype	N (%) ^a^	25(OH)D, nmol/L geometric mean (95%CI)	P_trend_ ^b^
CYP2R1 rs10741657	A/A	273 (21.2%)	56.5 (54.4, 58.8)	< 0.0001
	G/A	658 (51.0%)	51.8 (50.5, 53.2)	
	G/G	359 (27.8%)	49.8 (48.3, 51.5)	
CYP24A1 rs6013897	T/T	803 (62.3%)	52.9 (51.6, 54.1)	0.2001
	T/A	429 (33.3%)	51.3 (49.8, 52.9)	
	A/A	58 (4.5%)	50.0 (45.8, 54.6)	
DHCR7 rs12785878	A/A	519 (40.2%)	54.1 (52.6, 55.7)	0.0024
	A/C	592 (45.8%)	51.5 (50.1, 52.9)	
	C/C	181 (14.0%)	49.4 (47.1, 51. 8)	
DHCR7 rs1790349	A/A	678 (52.5%)	54.1 (52.8, 55.5)	0.0002
	A/G	515 (39.9%)	50.2 (48.7, 51.7)	
	G/G	99 (7.7%)	50.0 (46.9, 53.3)	
GC/VDBP rs2282679	A/A	670 (51.9%)	54.9 (53.6, 56.3)	< 0.0001
	A/C	525 (40.7%)	49. 7 (48.3, 51.1)	
	C/C	95 (7.4%)	48.4 (45.1, 52.0)	
GC/VDBP rs1155563	A/A	657 (51.3%)	54.7 (53.3, 56.0)	< 0.0001
	A/G	513 (40.0%)	49. 6 (48.1, 51.1)	
	G/G	112 (8.7%)	50.9 (47.9, 54.1)	
GC/VDBP rs7041	C/C	479 (37.1%)	55.9 (54.3, 57.5)	< 0.0001
	C/A	599 (46.4%)	50.9 (49.6, 52.3)	
	A/A	212 (16.4%)	48.1 (46.0, 50.3)	

a. Number of controls for each genotype with measured 25(OH)D

b. P value for trend was calculated by ANOVA

Though p-values were <0.05 for some tag SNPs (S2 Table) and haplotypes (S3 Table) in VDR, CYP24A1, and RXRA prior to FDR adjustment, none of the SNPs or haplotypes were significantly associated with breast cancer risk after accounting for FDR. We did not observe differences in the SNP-breast cancer risk associations according to menopausal status of the study subjects or estrogen receptor status of the breast tumors (data not shown).

## Discussion

In this nested case-control study among Swedish women, we confirmed the association of SNPs in CYP2R1, DHCR7, and GC/VDBP with 25(OH)D concentrations that were reported by GWAS [26, 27]. These SNPs, though, were not significantly associated with breast cancer risk, nor were the other SNPs or haplotypes that we examined in key vitamin D-related genes (VDR, CYP24A1, RXRA, and CYP27B1). Tag SNPs were used to capture genetic variation across three genes (as well as the 10 kb regions upstream and downstream of the genes). Though tag SNPs may not capture all of the genetic variation of a given gene, the use of an r^2^ of 0.8 for the selection of tags as well as the inclusion of multiple SNPs in high LD within large LD blocks in our study provides good coverage. We evaluated haplotypes in VDR, RXRA, and CYP24A1 only for LD blocks (defined using 1000 Genomes data) that included at least three SNPs that were genotyped in our study. This is not a comprehensive assessment of haplotypes across all of the genes for which tag SNPs were genotyped. However we were able to assess associations for several large LD blocks for which we genotyped multiple SNPs per block and did not observe any association. A limitation of our study is the fairly small size leading to limited power to detect associations. We had 80% power to detect ORs 1.2–1.3 for SNPs with MAF of 15–49% (n = 78 SNPs) and ORs between 1.3–1.5 for SNPs with MAF 5–15% (n = 30 SNPs). However, our results are generally in agreement with those of previous studies, in which the associations between most candidate SNPs in vitamin D-related genes and risk of breast cancer have been null or mixed [14–17, 19]. No previous studies have examined RXRA SNPs and breast cancer risk, and only a few studies have examined CYP24A1 SNPs [18–20, 22–25], with inconsistent results.

## Conclusions

After taking into account the false discovery rate, we did not observe any statistically significant association between SNPs in key vitamin D-related genes and risk of breast cancer. Our results, combined with the lack of association of 25(OH)D with risk reported by most prospective studies, do not support the hypothesis that vitamin D is associated with breast cancer risk [3].

## Supporting Information

S1 Fig LD heatmaps for VDR, RXRA, and CYP24A1, NSMSC controls and 1000 genomes. (JPG)

S1 Table Odds ratios for breast cancer and 95% confidence intervals of SNPs associated with 25(OH)D concentration. (DOCX)

S2 Table Odds ratios for breast cancer and 95% confidence intervals of tag SNPs in vitamin D-related genes. (DOCX)

S3 Table Haplotypes of VDR, RXRA, and CYP24A1 and risk of breast cancer. (DOCX)
