# Supplementary figures and images for: Genetic Polymorphisms in Vitamin D Metabolism and Signaling Genes and Risk of Breast Cancer: A Nested Case-Control Study
Source: PLoS One. 2015 Oct 21;10(10):e0140478. doi: 10.1371/journal.pone.0140478 (PMC4619526; doi:10.1371/journal.pone.0140478)

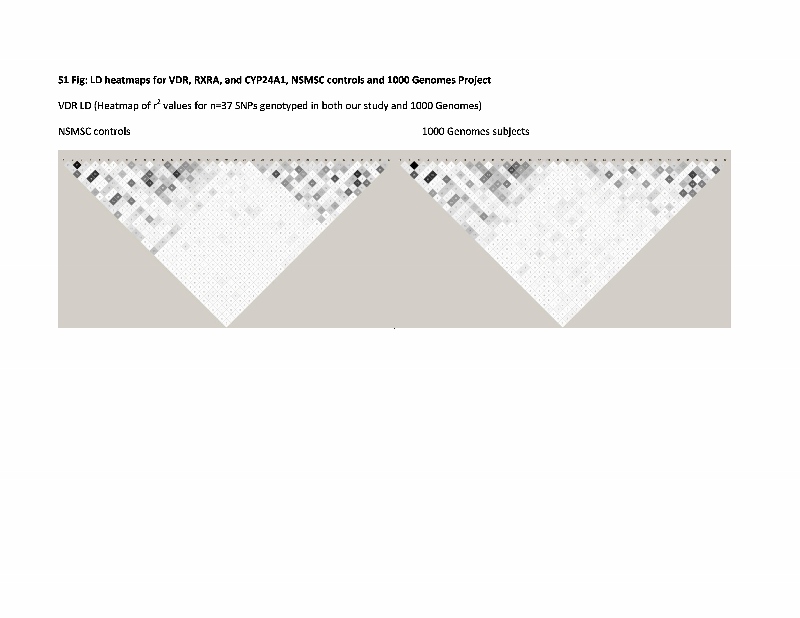

Supplement: S1 Fig — (JPG) [file pone.0140478.s001.jpg]
