# Supplementary material for: Genetic Polymorphisms in Vitamin D Metabolism and Signaling Genes and Risk of Breast Cancer: A Nested Case-Control Study
Source: PLoS One. 2015 Oct 21;10(10):e0140478. doi: 10.1371/journal.pone.0140478 (PMC4619526; doi:10.1371/journal.pone.0140478)
Supplement: S1 Table — (DOCX) [file pone.0140478.s002.docx]

S1 Table. Odds ratios for breast cancer and 95% confidence intervals of SNPs associated with 25(OH)D concentration

| Gene/SNP | Genotype | Cases,  n (%) | Controls,  n (%) | Age- and menopausal status-adjusted model^a^ | Multivariate-adjusted model^b^ | | |
| --- | --- | --- | --- | --- | --- | --- | --- |
|  |  |  |  | OR (95%CI) | OR (95%CI) | P_trend_^c^ | FDR^d^ |
| CYP2R1 rs10741657 | A/A | 175 (23.9%) | 307 (21.4%) | Ref. | Ref. | 0.26 | 0.62 |
|  | A/G | 358 (48.8%) | 720 (50.3%) | 1.00 (0.81, 1.24) | 0.95 (0.84, 1.08) |  |  |
|  | G/G | 200 (27.3%) | 405 (28.3%) | 1.16 (0.90, 1.49) | 0.95 (0.83, 1.09) |  |  |
| CYP24A1 rs6013897 | T/T | 473 (64.5%) | 888 (62.1%) | Ref. | Ref. | 0.58 | 0.81 |
|  | T/A | 223 (30.4%) | 477 (33.3%) | 0.88 (0.73, 1.07) | 0.91 (0.76, 1.09) |  |  |
|  | A/A | 37 (5.0%) | 66 (4.6%) | 1.06 (0.70, 1.61) | 1.08 (0.81, 1.43) |  |  |
| DHCR7 rs12785878 | A/A | 273 (37.2%) | 571 (39.8%) | Ref. | Ref. | 0.92 | 0.97 |
|  | A/C | 371 (50.6%) | 659 (46.0%) | 1.18 (0.97, 1.43) | 1.14 (1.00, 1.30) |  |  |
|  | C/C | 89 (12.1%) | 203 (14.2%) | 0.92 (0.69, 1.22) | 0.90 (0.75, 1.08) |  |  |
| DHCR7 rs1790349 | A/A | 348 (47.5%) | 752 (52.6%) | Ref. | Ref. | 0.11 | 0.46 |
|  | A/G | 326 (44.5%) | 571 (39.9%) | 1.22 (1.01, 1.46) | 1.08 (0.93, 1.25) |  |  |
|  | G/G | 58 (7.9%) | 108 (7.5%) | 1.16 (0.82, 1.64) | 1.03 (0.82, 1.29) |  |  |
| GC/VDBP rs2282679 | A/A | 381 (52.0%) | 754 (52.7%) | Ref. | Ref. | 0.72 | 0.89 |
|  | A/C | 293 (40.0%) | 571 (39.9%) | 1.03 (0.85, 1.24) | 0.97 (0.84, 1.13) |  |  |
|  | C/C | 59 (8.0%) | 106 (7.4%) | 1.12 (0.80, 1.57) | 1.06 (0.84, 1.32) |  |  |
| GC/VDBP rs1155563 | A/A | 379 (52.0%) | 737 (52.1%) | Ref. | Ref. | 0.52 | 0.81 |
|  | A/G | 298 (40.9%) | 553 (39.1%) | 1.05 (0.87, 1.27) | 1.11 (0.95, 1.29) |  |  |
|  | G/G | 52 (7.1%) | 125 (8.8%) | 0.81 (0.57, 1.15) | 0.84 (0.67, 1.06) |  |  |
| GC/VDBP rs7041 | C/C | 265 (36.1%) | 546 (38.1%) | Ref. | Ref. | 0.37 | 0.72 |
|  | C/A | 348 (47.4%) | 658 (45.9%) | 1.11 (0.91, 1.35) | 1.06 (0.93, 1.20) |  |  |
|  | A/A | 121 (16.5%) | 229 (16.0%) | 1.10 (0.85, 1.44) | 1.02 (0.86, 1.20) |  |  |

a. Conditional logistic regression, matching factors: age and menopausal status at blood donation (partial matching)

b. Conditional logistic regression, matching factors: age and menopausal status at blood donation (partial matching), covariates: age at menarche (continuous), family history of breast cancer (yes/no), BMI (log transformed), hormone replacement therapy (HRT, ever/never), and full term pregnancy (ordered, age at first full term pregnancy ≤20 years, 21-25 years, 26-30 years, > 30 years, nulliparous). Missing data for the following covariates was imputed using fully conditional specification multiple imputation: age at menarche, BMI, HRT, and age at first full term pregnancy.

c. P values for trend were calculated with 0, 1, 2 (or 0, 1) corresponding to the genotypes and were two-sided.

d. P values for trend were calculated with 0, 1, 2 (or 0, 1) corresponding to the genotypes and were two-sided. False discovery rate calculated by using the linear step-up method of Benjamini and Hochberg in SAS (PROC MULTTEST)
