# Supplementary material for: Genetic Polymorphisms in Vitamin D Metabolism and Signaling Genes and Risk of Breast Cancer: A Nested Case-Control Study
Source: PLoS One. 2015 Oct 21;10(10):e0140478. doi: 10.1371/journal.pone.0140478 (PMC4619526; doi:10.1371/journal.pone.0140478)
Supplement: S2 Table — (DOCX) [file pone.0140478.s003.docx]

S2 Table. Odds ratios for breast cancer and 95% confidence intervals of tag SNPs in vitamin D-related genes

| Gene/SNP | Genotype | Cases,  n (%) | Controls,  n (%) | Age- and menopausal status-adjusted model^a^ |  | | Multivariate-adjusted model^b^ | | |
| --- | --- | --- | --- | --- | --- | --- | --- | --- | --- |
|  |  |  |  | OR (95%CI) |  | OR (95%CI) | | P_trend_^c^ | FDR^d^ |
| **VDR** |  |  |  |  |  |  | |  |  |
| VDR rs7965281 | G/G | 230 (31.5%) | 398 (27.9%) | Ref. |  | Ref. | | 0.02 | 0.20 |
|  | G/A | 377 (51.6%) | 738 (51.7%) | 0.87 (0.71, 1.08) |  | 1.00 (0.89, 1.14) | |  |  |
|  | A/A | 124 (17.0%) | 292 (20.4%) | 0.73 (0.56, 0.95) |  | 0.85 (0.72, 0.99) | |  |  |
| VDR rs12581281 | G/G | 710 (96.9%) | 1364 (95.2%) | Ref. |  | Ref. | | 0.02 | 0.20 |
|  | A/G or A/A | 23 (3.1%) | 69 (4.8%) | 0.62 (0.38, 1.01) |  | 0.74 (0.58, 0.96) | |  |  |
| VDR rs4237856 | A/A | 385 (52.5%) | 839 (58.6%) | Ref. |  | Ref. | | 0.03 | 0.23 |
|  | A/C | 296 (40.4%) | 507 (35.4%) | 1.26 (1.05, 1.52) |  | 1.07 (0.91, 1.25) | |  |  |
|  | C/C | 52 (7.1%) | 86 (6.0%) | 1.30 (0.90, 1.87) |  | 1.09 (0.85, 1.39) | |  |  |
| VDR rs4760655 | A/A | 303 (41.3%) | 642 (44.8%) | Ref. |  | Ref. | | 0.04 | 0.26 |
|  | A/G | 328 (44.7%) | 637 (44.5%) | 1.07 (0.89, 1.30) |  | 0.94 (0.82, 1.07) | |  |  |
|  | G/G | 103 (14.0%) | 154 (10.7%) | 1.38 (1.04, 1.82) |  | 1.22 (1.02, 1.46) | |  |  |
| VDR rs9729 | C/C | 125 (17.2%) | 280 (19.7%) | Ref. |  | Ref. | | 0.05 | 0.32 |
|  | A/C | 371 (51.0%) | 729 (51.4%) | 1.14 (0.89, 1.46) |  | 1.00 (0.88, 1.13) | |  |  |
|  | A/A | 232 (31.9%) | 409 (28.8%) | 1.28 (0.98, 1.67) |  | 1.14 (0.99, 1.31) | |  |  |
| VDR rs11168267 | G/G | 543 (74.0%) | 1110 (77.4%) | Ref. |  | Ref. | | 0.06 | 0.32 |
|  | A/G | 179 (24.4%) | 308 (21.5%) | 1.20 (0.96, 1.48) |  | 0.96 (0.72, 1.28) | |  |  |
|  | A/A | 12 (1.6%) | 16 (1.1%) | 1.58 (0.73, 3.41) |  | 1.30 (0.77, 2.21) | |  |  |
| VDR rs11574050 | G/G | 628 (85.7%) | 1255 (87.6%) | Ref. |  | Ref. | | 0.07 | 0.38 |
|  | A/G | 97 (13.2%) | 172 (12.0%) | 1.13 (0.86, 1.48) |  | 0.75 (0.50, 1.11) | |  |  |
|  | A/A | 8 (1.1%) | 6 (0.4%) | 2.73 (0.94, 7.87) |  | 2.08 (1.00, 4.32) | |  |  |
| VDR rs12721364 | G/G | 551 (75.3%) | 1028 (71.9%) | Ref. |  | Ref. | | 0.09 | 0.42 |
|  | G/A | 168 (23.0%) | 367 (25.7%) | 0.85 (0.69, 1.05) |  | 1.00 (0.78, 1.29) | |  |  |
|  | A/A | 13 (1.8%) | 34 (2.4%) | 0.70 (0.37, 1.33) |  | 0.85 (0.55, 1.32) | |  |  |
| VDR rs7299460 | G/G | 396 (54.0%) | 748 (52.3%) | Ref. |  | Ref. | | 0.10 | 0.42 |
|  | G/A | 285 (38.9%) | 542 (37.9%) | 0.99 (0.82, 1.19) |  | 1.14 (0.98, 1.33) | |  |  |
|  | A/A | 52 (7.1%) | 141 (9.9%) | 0.69 (0.49, 0.98) |  | 0.76 (0.61, 0.96) | |  |  |
| VDR rs3847987 | C/C | 507 (70.1%) | 1021 (73.0%) | Ref. |  | Ref. | | 0.13 | 0.49 |
|  | C/A | 198 (27.4%) | 350 (25.0%) | 1.14 (0.93, 1.41) |  | 1.02 (0.80, 1.30) | |  |  |
|  | A/A | 18 (2.5%) | 27 (1.9%) | 1.32 (0.72, 2.43) |  | 1.12 (0.73, 1.70) | |  |  |
| VDR rs757343 | G/G | 515 (70.5%) | 1038 (73.1%) | Ref. |  | Ref. | | 0.13 | 0.49 |
|  | G/A | 196 (26.8%) | 357 (25.1%) | 1.12 (0.91, 1.38) |  | 0.94 (0.74, 1.19) | |  |  |
|  | A/A | 20 (2.7%) | 25 (1.8%) | 1.59 (0.87, 2.90) |  | 1.27 (0.84, 1.92) | |  |  |
| VDR rs2238136 | G/G | 389 (53.1%) | 802 (56.0%) | Ref. |  | Ref. | | 0.14 | 0.49 |
|  | G/A | 278 (37.9%) | 525 (36.7%) | 1.09 (0.90, 1.31) |  | 0.97 (0.84, 1.13) | |  |  |
|  | A/A | 66 (9.0%) | 105 (7.3%) | 1.28 (0.92, 1.79) |  | 1.15 (0.92, 1.43) | |  |  |
| VDR rs2239179 | A/A | 232 (31.7%) | 489 (34.3%) | Ref. |  | Ref. | | 0.15 | 0.49 |
|  | A/G | 372 (50.9%) | 709 (49.8%) | 1.10 (0.90, 1.35) |  | 1.02 (0.90, 1.15) | |  |  |
|  | G/G | 127 (17.4%) | 226 (15.9%) | 1.19 (0.91, 1.55) |  | 1.09 (0.93, 1.28) | |  |  |
| VDR rs3819545 | A/A | 290 (39.5%) | 528 (36.8%) | Ref. |  | Ref. | | 0.16 | 0.49 |
|  | A/G | 343 (46.7%) | 691 (48.2%) | 0.90 (0.74, 1.10) |  | 0.98 (0.86, 1.12) | |  |  |
|  | G/G | 101 (13.8%) | 215 (15.0%) | 0.85 (0.64, 1.12) |  | 0.92 (0.77, 1.10) | |  |  |
| VDR rs1540339 | G/G | 264 (36.1%) | 485 (34.1%) | Ref. |  | Ref. | | 0.17 | 0.49 |
|  | G/A | 359 (49.0%) | 698 (49.1%) | 0.95 (0.78, 1.16) |  | 1.04 (0.91, 1.18) | |  |  |
|  | A/A | 109 (14.9%) | 239 (16.8%) | 0.84 (0.64, 1.10) |  | 0.88 (0.75, 1.05) | |  |  |
| VDR rs11168287 | A/A | 228 (31.1%) | 495 (34.5%) | Ref. |  | Ref. | | 0.19 | 0.53 |
|  | A/G | 368 (50.1%) | 684 (47.7%) | 1.16 (0.95, 1.41) |  | 1.04 (0.91, 1.17) | |  |  |
|  | G/G | 138 (18.8%) | 256 (17.8%) | 1.15 (0.89, 1.48) |  | 1.06 (0.91, 1.24) | |  |  |
| VDR rs4547172 | G/G | 691 (94.8%) | 1321 (93.4%) | Ref. |  | Ref. | | 0.21 | 0.56 |
|  | G/A | 38 (5.2%) | 93 (6.6%) | 0.78 (0.53, 1.15) |  | 0.88 (0.72, 1.08) | |  |  |
| VDR rs11574044 | A/A | 523 (71.4%) | 1002 (69.9%) | Ref. |  | Ref. | | 0.29 | 0.63 |
|  | A/C | 193 (26.3%) | 382 (26.7%) | 0.97 (0.79, 1.19) |  | 1.14 (0.91, 1.42) | |  |  |
|  | C/C | 17 (2.3%) | 49 (3.4%) | 0.66 (0.37, 1.16) |  | 0.75 (0.51, 1.10) | |  |  |
| VDR rs10875695 | C/C | 431 (58.8%) | 826 (57.6%) | Ref. |  | Ref. | | 0.29 | 0.63 |
|  | A/C | 260 (35.5%) | 499 (34.8%) | 1.00 (0.83, 1.21) |  | 1.11 (0.94, 1.30) | |  |  |
|  | A/A | 42 (5.7%) | 108 (7.5%) | 0.75 (0.52, 1.09) |  | 0.82 (0.63, 1.05) | |  |  |
| VDR rs1544410 (Bsm1) | G/G | 237 (32.3%) | 496 (34.9%) | Ref. |  | Ref. | | 0.29 | 0.63 |
|  | G/A | 383 (52.3%) | 709 (49.9%) | 1.14 (0.93, 1.39) |  | 1.06 (0.93, 1.21) | |  |  |
|  | A/A | 113 (15.4%) | 217 (15.3%) | 1.11 (0.84, 1.46) |  | 1.03 (0.86, 1.22) | |  |  |
| VDR rs7970376 | A/A | 170 (23.2%) | 375 (26.2%) | Ref. |  | Ref. | | 0.34 | 0.68 |
|  | A/G | 378 (51.5%) | 703 (49.0%) | 1.18 (0.95, 1.47) |  | 1.05 (0.93, 1.19) | |  |  |
|  | G/G | 186 (25.3%) | 356 (24.8%) | 1.13 (0.88, 1.46) |  | 1.04 (0.90, 1.20) | |  |  |
| VDR rs2239186 | A/A | 476 (64.9%) | 912 (63.7%) | Ref. |  | Ref. | | 0.34 | 0.68 |
|  | A/G | 227 (31.0%) | 451 (31.5%) | 0.97 (0.80, 1.18) |  | 1.05 (0.87, 1.27) | |  |  |
|  | G/G | 30 (4.1%) | 68 (4.8%) | 0.82 (0.52, 1.29) |  | 0.86 (0.63, 1.17) | |  |  |
| VDR rs2239180 | G/G | 534 (72.9%) | 1070 (74.8%) | Ref. |  | Ref. | | 0.35 | 0.69 |
|  | G/C | 184 (25.1%) | 331 (23.1%) | 1.11 (0.90, 1.38) |  | 1.02 (0.80, 1.32) | |  |  |
|  | C/C | 15 (2.0%) | 30 (2.1%) | 0.99 (0.52, 1.87) |  | 1.05 (0.68, 1.63) | |  |  |
| VDR rs2239182 | A/A | 202 (27.5%) | 398 (27.8%) | Ref. |  | Ref. | | 0.39 | 0.74 |
|  | A/G | 363 (49.5%) | 733 (51.2%) | 0.97 (0.78, 1.20) |  | 0.96 (0.85, 1.09) | |  |  |
|  | G/G | 169 (23.0%) | 300 (21.0%) | 1.10 (0.85, 1.42) |  | 1.08 (0.93, 1.25) | |  |  |
| VDR rs11574032 | G/G | 624 (85.0%) | 1239 (86.6%) | Ref. |  | Ref. | | 0.42 | 0.77 |
|  | A/G | 103 (14.0%) | 185 (12.9%) | 1.09 (0.85, 1.42) |  | 0.84 (0.56, 1.27) | |  |  |
|  | A/A | 7 (1.0%) | 7 (0.5%) | 2.05 (0.68, 6.22) |  | 1.48 (0.70, 3.13) | |  |  |
| VDR rs11568820 (Cdx2) | G/G | 500 (68.2%) | 962 (67.1%) | Ref. |  | Ref. | | 0.43 | 0.77 |
|  | A/G | 212 (28.9%) | 414 (28.9%) | 0.98 (0.80, 1.20) |  | 1.15 (0.93, 1.41) | |  |  |
|  | A/A | 21 (2.9%) | 57 (4.0%) | 0.71 (0.43, 1.20) |  | 0.77 (0.54, 1.09) | |  |  |
| VDR rs2408876 | A/A | 256 (34.9%) | 475 (33.2%) | Ref. |  | Ref. | | 0.45 | 0.78 |
|  | A/G | 342 (46.6%) | 685 (47.8%) | 0.93 (0.76, 1.14) |  | 0.97 (0.85, 1.10) | |  |  |
|  | G/G | 136 (18.5%) | 272 (19.0%) | 0.93 (0.72, 1.20) |  | 0.97 (0.83, 1.14) | |  |  |
| VDR rs2189480 | C/C | 337 (45.9%) | 644 (44.9%) | Ref. |  | Ref. | | 0.46 | 0.78 |
|  | C/A | 320 (43.6%) | 625 (43.6%) | 0.98 (0.81, 1.19) |  | 1.04 (0.90, 1.19) | |  |  |
|  | A/A | 77 (10.5%) | 166 (11.6%) | 0.89 (0.65, 1.20) |  | 0.91 (0.75, 1.11) | |  |  |
| VDR rs11574026 | G/G | 626 (85.3%) | 1235 (86.3%) | Ref. |  | Ref. | | 0.55 | 0.81 |
|  | A/G | 106 (14.4%) | 185 (12.9%) | 1.13 (0.88, 1.47) |  | 1.52 (0.89, 2.58) | |  |  |
|  | A/A | 2 (0.3%) | 11 (0.8%) | 0.37 (0.08, 1.68) |  | 0.51 (0.18, 1.41) | |  |  |
| VDR rs6580642 | G/G | 547 (74.5%) | 1056 (73.6%) | Ref. |  | Ref. | | 0.55 | 0.81 |
|  | G/A | 174 (23.7%) | 349 (24.3%) | 0.96 (0.78, 1.19) |  | 1.03 (0.79, 1.34) | |  |  |
|  | A/A | 13 (1.8%) | 29 (2.0%) | 0.85 (0.43, 1.67) |  | 0.90 (0.57, 1.42) | |  |  |
| VDR rs11574077 | A/A | 643 (87.8%) | 1268 (88.7%) | Ref. |  | Ref. | | 0.57 | 0.81 |
|  | A/G | 88 (12.0%) | 155 (10.8%) | 1.13 (0.86, 1.50) |  | 1.53 (0.74, 3.17) | |  |  |
|  | G/G | 1 (0.1%) | 7 (0.5%) | 0.29 (0.04, 2.36) |  | 0.49 (0.12, 2.00) | |  |  |
| VDR rs10875693 | T/T | 313 (43.1%) | 610 (43.2%) | Ref. |  | Ref. | | 0.57 | 0.81 |
|  | A/T | 319 (43.9%) | 641 (45.4%) | 0.97 (0.80, 1.18) |  | 0.93 (0.81, 1.06) | |  |  |
|  | A/A | 94 (12.9%) | 160 (11.3%) | 1.15 (0.86, 1.54) |  | 1.12 (0.93, 1.35) | |  |  |
| VDR rs2228570 (Fok 1) | G/G | 293 (39.9%) | 581 (40.5%) | Ref. |  | Ref. | | 0.58 | 0.81 |
|  | G/A | 330 (45.0%) | 647 (45.1%) | 1.01 (0.83, 1.22) |  | 0.99 (0.87, 1.13) | |  |  |
|  | A/A | 111 (15.1%) | 207 (14.4%) | 1.05 (0.80, 1.37) |  | 1.05 (0.88, 1.24) | |  |  |
| VDR rs2283342 | A/A | 559 (76.3%) | 1091 (76.1%) | Ref. |  | Ref. | | 0.66 | 0.86 |
|  | A/G | 161 (22.0%) | 316 (22.1%) | 1.00 (0.81, 1.24) |  | 1.04 (0.80, 1.36) | |  |  |
|  | G/G | 13 (1.8%) | 26 (1.8%) | 0.92 (0.46, 1.84) |  | 0.90 (0.56, 1.43) | |  |  |
| VDR rs4760648 | G/G | 267 (37.0%) | 503 (36.3%) | Ref. |  | Ref. | | 0.71 | 0.88 |
|  | G/A | 338 (46.8%) | 660 (47.6%) | 0.96 (0.79, 1.16) |  | 0.97 (0.85, 1.10) | |  |  |
|  | A/A | 117 (16.2%) | 224 (16.1%) | 0.99 (0.75, 1.29) |  | 1.00 (0.85, 1.18) | |  |  |
| VDR rs7139166 | C/C | 215 (29.3%) | 406 (28.4%) | Ref. |  | Ref. | | 0.71 | 0.88 |
|  | C/G | 366 (49.9%) | 714 (49.9%) | 0.98 (0.80, 1.22) |  | 0.99 (0.88, 1.12) | |  |  |
|  | G/G | 152 (20.7%) | 311 (21.7%) | 0.94 (0.73, 1.22) |  | 0.98 (0.85, 1.14) | |  |  |
| VDR rs3890733 | G/G | 297 (40.6%) | 550 (38.7%) | Ref. |  | Ref. | | 0.74 | 0.90 |
|  | G/A | 324 (44.3%) | 659 (46.4%) | 0.92 (0.76, 1.12) |  | 0.95 (0.83, 1.08) | |  |  |
|  | A/A | 110 (15.0%) | 212 (14.9%) | 0.98 (0.75, 1.28) |  | 1.02 (0.86, 1.21) | |  |  |
| VDR rs2254210 | G/G | 237 (32.3%) | 422 (29.4%) | Ref. |  | Ref. | | 0.79 | 0.94 |
|  | G/A | 353 (48.1%) | 748 (52.2%) | 0.85 (0.70, 1.04) |  | 0.90 (0.79, 1.02) | |  |  |
|  | A/A | 144 (19.6%) | 263 (18.4%) | 0.98 (0.76, 1.26) |  | 1.06 (0.91, 1.23) | |  |  |
| VDR rs11168275 | A/A | 385 (52.5%) | 753 (52.6%) | Ref. |  | Ref. | | 0.82 | 0.95 |
|  | A/G | 276 (37.7%) | 548 (38.3%) | 0.99 (0.82, 1.19) |  | 0.96 (0.83, 1.10) | |  |  |
|  | G/G | 72 (9.8%) | 131 (9.1%) | 1.08 (0.80, 1.48) |  | 1.07 (0.87, 1.31) | |  |  |
| VDR rs11574085 | A/A | 655 (89.2%) | 1270 (88.5%) | Ref. |  | Ref. | | 0.90 | 0.97 |
|  | A/T | 76 (10.4%) | 160 (11.1%) | 0.93 (0.70, 1.24) |  | 0.90 (0.53, 1.52) | |  |  |
|  | T/T | 3 (0.4%) | 5 (0.3%) | 1.09 (0.26, 4.61) |  | 1.20 (0.45, 3.20) | |  |  |
| VDR rs2107301 | G/G | 317 (43.4%) | 627 (44.3%) | Ref. |  | Ref. | | 0.92 | 0.97 |
|  | G/A | 336 (46.0%) | 628 (44.4%) | 1.05 (0.87, 1.27) |  | 1.06 (0.92, 1.21) | |  |  |
|  | A/A | 78 (10.7%) | 161 (11.4%) | 0.97 (0.71, 1.31) |  | 0.95 (0.78, 1.16) | |  |  |
| VDR rs2853564 | A/A | 249 (34.0%) | 466 (32.5%) | Ref. |  | Ref. | | 0.93 | 0.97 |
|  | A/G | 350 (47.7%) | 714 (49.9%) | 0.92 (0.75, 1.12) |  | 0.94 (0.83, 1.06) | |  |  |
|  | G/G | 134 (18.3%) | 252 (17.6%) | 1.00 (0.77, 1.29) |  | 1.04 (0.89, 1.22) | |  |  |
| **CYP24A1** |  |  |  |  |  |  | |  |  |
| CYP24A1 rs6068821 | G/G | 334 (45.5%) | 570 (39.8%) | Ref. |  | Ref. | | < 0.01 | 0.06 |
|  | G/A | 327 (44.6%) | 658 (45.9%) | 0.85 (0.70, 1.02) |  | 1.05 (0.91, 1.20) | |  |  |
|  | A/A | 73 (9.9%) | 205 (14.3%) | 0.60 (0.45, 0.82) |  | 0.75 (0.62, 0.92) | |  |  |
| CYP24A1 rs2208588 | T/T | 312 (42.6%) | 526 (36.9%) | Ref. |  | Ref. | | < 0.01 | 0.06 |
|  | T/A | 334 (45.6%) | 673 (47.2%) | 0.84 (0.70, 1.02) |  | 1.03 (0.90, 1.17) | |  |  |
|  | A/A | 86 (11.7%) | 226 (15.9%) | 0.63 (0.47, 0.84) |  | 0.78 (0.65, 0.93) | |  |  |
| CYP24A1 rs2181874 | G/G | 486 (66.3%) | 888 (62.0%) | Ref. |  | Ref. | | 0.01 | 0.20 |
|  | G/A | 217 (29.6%) | 470 (32.8%) | 0.84 (0.69, 1.02) |  | 0.98 (0.81, 1.18) | |  |  |
|  | A/A | 30 (4.1%) | 75 (5.2%) | 0.74 (0.47, 1.15) |  | 0.84 (0.62, 1.14) | |  |  |
| CYP24A1 rs2248359 | G/G | 409 (55.7%) | 708 (49.4%) | Ref. |  | Ref. | | 0.01 | 0.13 |
|  | G/A | 270 (36.8%) | 603 (42.1%) | 0.77 (0.64, 0.93) |  | 0.92 (0.79, 1.07) | |  |  |
|  | A/A | 55 (7.5%) | 122 (8.5%) | 0.76 (0.54, 1.08) |  | 0.89 (0.71, 1.12) | |  |  |
| CYP24A1 rs2762926 | C/C | 253 (34.7%) | 426 (29.9%) | Ref. |  | Ref. | | 0.01 | 0.13 |
|  | C/A | 353 (48.4%) | 709 (49.8%) | 0.84 (0.68, 1.03) |  | 0.99 (0.87, 1.12) | |  |  |
|  | A/A | 124 (17.0%) | 289 (20.3%) | 0.72 (0.56, 0.93) |  | 0.84 (0.71, 0.98) | |  |  |
| CYP24A1 rs2762929 | A/A | 277 (37.7%) | 473 (33.0%) | Ref. |  | Ref. | | 0.01 | 0.19 |
|  | A/G | 347 (47.3%) | 713 (49.7%) | 0.83 (0.68, 1.01) |  | 0.97 (0.86, 1.10) | |  |  |
|  | G/G | 110 (15.0%) | 249 (17.4%) | 0.76 (0.58, 0.99) |  | 0.86 (0.73, 1.01) | |  |  |
| CYP24A1 rs2296241 | A/A | 185 (25.2%) | 408 (28.5%) | Ref. |  | Ref. | | 0.01 | 0.13 |
|  | A/G | 358 (48.8%) | 719 (50.1%) | 1.10 (0.88, 1.37) |  | 0.95 (0.84, 1.08) | |  |  |
|  | G/G | 191 (26.0%) | 307 (21.4%) | 1.37 (1.07, 1.76) |  | 1.23 (1.07, 1.42) | |  |  |
| CYP24A1 rs2762927 | C/C | 316 (43.3%) | 562 (39.5%) | Ref. |  | Ref. | | 0.02 | 0.20 |
|  | C/A | 335 (45.9%) | 671 (47.2%) | 0.89 (0.73, 1.07) |  | 1.01 (0.88, 1.15) | |  |  |
|  | A/A | 79 (10.8%) | 188 (13.2%) | 0.76 (0.56, 1.01) |  | 0.84 (0.70, 1.02) | |  |  |
| CYP24A1 rs2585413 | G/G | 384 (52.5%) | 702 (49.2%) | Ref. |  | Ref. | | 0.02 | 0.20 |
|  | G/A | 301 (41.2%) | 590 (41.4%) | 0.94 (0.78, 1.13) |  | 1.11 (0.95, 1.30) | |  |  |
|  | A/A | 46 (6.3%) | 134 (9.4%) | 0.64 (0.45, 0.91) |  | 0.74 (0.59, 0.94) | |  |  |
| CYP24A1 rs927650 | G/G | 183 (25.1%) | 401 (28.3%) | Ref. |  | Ref. | | 0.02 | 0.20 |
|  | G/A | 371 (50.8%) | 724 (51.1%) | 1.12 (0.90, 1.40) |  | 1.00 (0.88, 1.13) | |  |  |
|  | A/A | 176 (24.1%) | 291 (20.6%) | 1.31 (1.01, 1.68) |  | 1.17 (1.01, 1.35) | |  |  |
| CYP24A1 rs2762934 | G/G | 512 (69.8%) | 950 (66.4%) | Ref. |  | Ref. | | 0.03 | 0.25 |
|  | G/A | 197 (26.9%) | 425 (29.7%) | 0.85 (0.70, 1.05) |  | 0.98 (0.80, 1.21) | |  |  |
|  | A/A | 24 (3.3%) | 55 (3.8%) | 0.81 (0.50, 1.33) |  | 0.85 (0.61, 1.20) | |  |  |
| CYP24A1 rs4809960 | A/A | 479 (65.3%) | 861 (60.1%) | Ref. |  | Ref. | | 0.04 | 0.29 |
|  | A/G | 218 (29.7%) | 496 (34.6%) | 0.79 (0.65, 0.96) |  | 0.88 (0.73, 1.05) | |  |  |
|  | G/G | 36 (4.9%) | 76 (5.3%) | 0.86 (0.57, 1.29) |  | 1.01 (0.76, 1.34) | |  |  |
| CYP24A1 rs6022999 | A/A | 479 (65.3%) | 888 (61.9%) | Ref. |  | Ref. | | 0.05 | 0.31 |
|  | A/G | 229 (31.2%) | 481 (33.5%) | 0.89 (0.73, 1.07) |  | 1.01 (0.83, 1.22) | |  |  |
|  | G/G | 26 (3.5%) | 66 (4.6%) | 0.71 (0.44, 1.14) |  | 0.84 (0.61, 1.16) | |  |  |
| CYP24A1 rs6127119 | G/G | 481 (65.7%) | 867 (60.8%) | Ref. |  | Ref. | | 0.07 | 0.36 |
|  | G/A | 213 (29.1%) | 479 (33.6%) | 0.80 (0.66, 0.97) |  | 0.94 (0.79, 1.12) | |  |  |
|  | A/A | 38 (5.2%) | 81 (5.7%) | 0.86 (0.57, 1.28) |  | 0.94 (0.71, 1.23) | |  |  |
| CYP24A1 rs2244719 | A/A | 169 (23.1%) | 368 (25.8%) | Ref. |  | Ref. | | 0.09 | 0.42 |
|  | G/A | 366 (50.0%) | 710 (49.7%) | 1.12 (0.90, 1.41) |  | 1.02 (0.90, 1.15) | |  |  |
|  | G/G | 197 (26.9%) | 351 (24.6%) | 1.22 (0.95, 1.57) |  | 1.11 (0.96, 1.28) | |  |  |
| CYP24A1 rs2762933 | T/T | 374 (51.1%) | 682 (48.1%) | Ref. |  | Ref. | | 0.09 | 0.42 |
|  | T/A | 297 (40.6%) | 602 (42.5%) | 0.90 (0.74, 1.09) |  | 1.01 (0.87, 1.17) | |  |  |
|  | A/A | 61 (8.3%) | 134 (9.4%) | 0.83 (0.59, 1.15) |  | 0.87 (0.70, 1.08) | |  |  |
| CYP24A1 rs2209314 | A/A | 414 (56.6%) | 773 (54.1%) | Ref. |  | Ref. | | 0.11 | 0.46 |
|  | A/G | 266 (36.3%) | 544 (38.0%) | 0.91 (0.75, 1.10) |  | 1.02 (0.87, 1.19) | |  |  |
|  | G/G | 52 (7.1%) | 113 (7.9%) | 0.86 (0.61, 1.23) |  | 0.87 (0.69, 1.10) | |  |  |
| CYP24A1 rs6022985 | G/G | 401 (54.9%) | 735 (51.9%) | Ref. |  | Ref. | | 0.14 | 0.49 |
|  | G/C | 278 (38.1%) | 559 (39.5%) | 0.91 (0.76, 1.10) |  | 1.01 (0.87, 1.17) | |  |  |
|  | C/C | 51 (7.0%) | 121 (8.6%) | 0.77 (0.54, 1.09) |  | 0.89 (0.70, 1.12) | |  |  |
| CYP24A1 rs2426498 | G/G | 589 (80.2%) | 1112 (77.6%) | Ref. |  | Ref. | | 0.15 | 0.49 |
|  | G/C | 137 (18.7%) | 300 (20.9%) | 0.85 (0.68, 1.07) |  | 1.04 (0.76, 1.43) | |  |  |
|  | C/C | 8 (1.1%) | 21 (1.5%) | 0.72 (0.32, 1.64) |  | 0.80 (0.46, 1.41) | |  |  |
| CYP24A1 rs2245153 | A/A | 525 (72.6%) | 969 (69.8%) | Ref. |  | Ref. | | 0.16 | 0.49 |
|  | A/G | 177 (24.5%) | 375 (27.0%) | 0.87 (0.70, 1.07) |  | 0.95 (0.76, 1.19) | |  |  |
|  | G/G | 21 (2.9%) | 45 (3.2%) | 0.84 (0.49, 1.44) |  | 0.95 (0.66, 1.37) | |  |  |
| CYP24A1 rs4809958 | A/A | 551 (75.1%) | 1032 (71.9%) | Ref. |  | Ref. | | 0.17 | 0.49 |
|  | A/C | 168 (22.9%) | 367 (25.6%) | 0.86 (0.70, 1.06) |  | 0.99 (0.78, 1.27) | |  |  |
|  | C/C | 15 (2.0%) | 36 (2.5%) | 0.79 (0.43, 1.46) |  | 0.89 (0.58, 1.36) | |  |  |
| CYP24A1 rs3787555 | C/C | 440 (60.4%) | 815 (57.5%) | Ref. |  | Ref. | | 0.19 | 0.53 |
|  | C/A | 247 (33.9%) | 509 (35.9%) | 0.90 (0.75, 1.09) |  | 1.00 (0.85, 1.18) | |  |  |
|  | A/A | 41 (5.6%) | 94 (6.6%) | 0.81 (0.55, 1.19) |  | 0.91 (0.70, 1.18) | |  |  |
| CYP24A1 rs6097801 | G/G | 566 (77.2%) | 1084 (75.7%) | Ref. |  | Ref. | | 0.23 | 0.60 |
|  | G/A | 161 (22.0%) | 316 (22.1%) | 0.98 (0.79, 1.21) |  | 1.32 (0.96, 1.82) | |  |  |
|  | A/A | 6 (0.8%) | 32 (2.2%) | 0.36 (0.15, 0.86) |  | 0.56 (0.31, 1.00) | |  |  |
| CYP24A1 rs2296239 | G/G | 452 (61.7%) | 847 (59.3%) | Ref. |  | Ref. | | 0.26 | 0.62 |
|  | G/A | 242 (33.1%) | 495 (34.7%) | 0.92 (0.76, 1.11) |  | 0.99 (0.84, 1.18) | |  |  |
|  | A/A | 38 (5.2%) | 86 (6.0%) | 0.82 (0.55, 1.21) |  | 0.92 (0.71, 1.20) | |  |  |
| CYP24A1 rs2585424 | C/C | 661 (90.3%) | 1259 (88.1%) | Ref. |  | Ref. | | 0.27 | 0.62 |
|  | C/A | 65 (8.9%) | 164 (11.5%) | 0.76 (0.56, 1.03) |  | 0.74 (0.48, 1.13) | |  |  |
|  | A/A | 6 (0.8%) | 6 (0.4%) | 1.79 (0.57, 5.62) |  | 1.44 (0.66, 3.13) | |  |  |
| CYP24A1 rs3787557 | A/A | 567 (77.2%) | 1075 (75.0%) | Ref. |  | Ref. | | 0.32 | 0.67 |
|  | A/G | 155 (21.1%) | 335 (23.4%) | 0.87 (0.70, 1.09) |  | 0.90 (0.68, 1.19) | |  |  |
|  | G/G | 12 (1.6%) | 24 (1.7%) | 0.96 (0.47, 1.95) |  | 1.07 (0.65, 1.74) | |  |  |
| CYP24A1 rs1570669 | A/A | 324 (44.3%) | 618 (43.2%) | Ref. |  | Ref. | | 0.46 | 0.78 |
|  | A/G | 322 (44.0%) | 619 (43.3%) | 0.99 (0.82, 1.20) |  | 1.05 (0.92, 1.20) | |  |  |
|  | G/G | 86 (11.7%) | 192 (13.4%) | 0.85 (0.64, 1.13) |  | 0.91 (0.75, 1.09) | |  |  |
| CYP24A1 rs2762941 | G/G | 301 (41.1%) | 583 (40.7%) | Ref. |  | Ref. | | 0.50 | 0.80 |
|  | G/A | 340 (46.4%) | 656 (45.8%) | 1.00 (0.82, 1.21) |  | 1.03 (0.90, 1.18) | |  |  |
|  | A/A | 92 (12.6%) | 192 (13.4%) | 0.92 (0.69, 1.23) |  | 0.93 (0.77, 1.12) | |  |  |
| CYP24A1 rs6068816 | G/G | 590 (80.5%) | 1149 (80.2%) | Ref. |  | Ref. | | 0.81 | 0.95 |
|  | A/G | 136 (18.6%) | 264 (18.4%) | 1.00 (0.80, 1.25) |  | 1.13 (0.82, 1.58) | |  |  |
|  | A/A | 7 (1.0%) | 19 (1.3%) | 0.71 (0.30, 1.71) |  | 0.79 (0.43, 1.43) | |  |  |
| CYP24A1 rs6097809 | A/A | 637 (87.3%) | 1246 (87.7%) | Ref. |  | Ref. | | 0.96 | 0.97 |
|  | A/G | 89 (12.2%) | 167 (11.8%) | 1.03 (0.78, 1.35) |  | 0.95 (0.60, 1.50) | |  |  |
|  | G/G | 4 (0.5%) | 8 (0.6%) | 1.00 (0.30, 3.32) |  | 1.08 (0.47, 2.49) | |  |  |
| **RXRA** |  |  |  |  |  |  | |  |  |
| RXRA rs12006409 | G/G | 597 (82.0%) | 1097 (78.1%) | Ref. |  | Ref. | | 0.03 | 0.23 |
|  | G/A | 126 (17.3%) | 289 (20.6%) | 0.80 (0.64, 1.01) |  | 1.04 (0.72, 1.49) | |  |  |
|  | A/A | 5 (0.7%) | 19 (1.4%) | 0.51 (0.19, 1.36) |  | 0.73 (0.37, 1.43) | |  |  |
| RXRA rs4917348 | A/A | 600 (83.4%) | 1188 (85.7%) | Ref. |  | Ref. | | 0.16 | 0.49 |
|  | A/G | 110 (15.3%) | 185 (13.3%) | 1.15 (0.89, 1.48) |  | 0.96 (0.69, 1.33) | |  |  |
|  | G/G | 9 (1.3%) | 13 (0.9%) | 1.38 (0.59, 3.23) |  | 1.27 (0.71, 2.27) | |  |  |
| RXRA rs11185644 | A/A | 577 (78.6%) | 1157 (80.6%) | Ref. |  | Ref. | | 0.17 | 0.49 |
|  | A/G | 146 (19.9%) | 264 (18.4%) | 1.10 (0.88, 1.38) |  | 0.88 (0.65, 1.20) | |  |  |
|  | G/G | 11 (1.5%) | 14 (1.0%) | 1.61 (0.73, 3.56) |  | 1.42 (0.83, 2.45) | |  |  |
| RXRA rs12339187 | A/A | 577 (79.0%) | 1103 (77.5%) | Ref. |  | Ref. | | 0.24 | 0.61 |
|  | A/G | 145 (19.9%) | 297 (20.9%) | 0.92 (0.74, 1.16) |  | 1.04 (0.77, 1.42) | |  |  |
|  | G/G | 8 (1.1%) | 23 (1.6%) | 0.67 (0.30, 1.50) |  | 0.83 (0.48, 1.43) | |  |  |
| RXRA rs35180144 | A/A | 594 (81.1%) | 1184 (82.7%) | Ref. |  | Ref. | | 0.25 | 0.62 |
|  | A/C | 129 (17.6%) | 236 (16.5%) | 1.10 (0.86, 1.39) |  | 0.92 (0.66, 1.29) | |  |  |
|  | C/C | 9 (1.2%) | 11 (0.8%) | 1.61 (0.67, 3.91) |  | 1.30 (0.71, 2.38) | |  |  |
| RXRA rs10881583 | A/A | 489 (66.7%) | 923 (64.5%) | Ref. |  | Ref. | | 0.27 | 0.62 |
|  | A/G | 220 (30.0%) | 449 (31.4%) | 0.91 (0.75, 1.11) |  | 1.01 (0.83, 1.23) | |  |  |
|  | G/G | 24 (3.3%) | 58 (4.1%) | 0.78 (0.48, 1.27) |  | 0.90 (0.65, 1.25) | |  |  |
| RXRA rs1110102 | C/C | 661 (90.4%) | 1303 (91.4%) | Ref. |  | Ref. | | 0.32 | 0.67 |
|  | C/G or G/G | 70 (9.6%) | 122 (8.6%) | 1.13 (0.83, 1.54) |  | 1.08 (0.92, 1.27) | |  |  |
| RXRA rs12004589 | C/C | 596 (81.9%) | 1130 (80.0%) | Ref. |  | Ref. | | 0.38 | 0.72 |
|  | C/A | 124 (17.0%) | 267 (18.9%) | 0.87 (0.69, 1.10) |  | 0.87 (0.62, 1.21) | |  |  |
|  | A/A | 8 (1.1%) | 15 (1.1%) | 1.05 (0.44, 2.54) |  | 1.15 (0.63, 2.10) | |  |  |
| RXRA rs34312136 | A/A | 218 (29.7%) | 458 (31.9%) | Ref. |  | Ref. | | 0.46 | 0.78 |
|  | A/G | 396 (54.0%) | 680 (47.4%) | 1.20 (0.98, 1.46) |  | 1.19 (1.06, 1.35) | |  |  |
|  | G/G | 120 (16.3%) | 296 (20.6%) | 0.84 (0.64, 1.10) |  | 0.84 (0.71, 0.99) | |  |  |
| RXRA rs2234753 | A/A | 438 (59.9%) | 901 (63.5%) | Ref. |  | Ref. | | 0.47 | 0.78 |
|  | A/G | 263 (36.0%) | 439 (31.0%) | 1.22 (1.00, 1.48) |  | 1.21 (1.01, 1.44) | |  |  |
|  | G/G | 30 (4.1%) | 78 (5.5%) | 0.79 (0.51, 1.22) |  | 0.82 (0.62, 1.10) | |  |  |
| RXRA rs9409929 | G/G | 343 (46.9%) | 656 (45.9%) | Ref. |  | Ref. | | 0.47 | 0.78 |
|  | G/A | 322 (44.0%) | 621 (43.5%) | 0.99 (0.82, 1.19) |  | 1.06 (0.93, 1.22) | |  |  |
|  | A/A | 67 (9.2%) | 151 (10.6%) | 0.85 (0.61, 1.17) |  | 0.89 (0.72, 1.09) | |  |  |
| RXRA rs34798391 | A/A | 549 (74.8%) | 1101 (76.7%) | Ref. |  | Ref. | | 0.50 | 0.80 |
|  | A/G | 177 (24.1%) | 317 (22.1%) | 1.12 (0.90, 1.38) |  | 1.12 (0.82, 1.54) | |  |  |
|  | G/G | 8 (1.1%) | 17 (1.2%) | 0.83 (0.36, 1.95) |  | 0.88 (0.49, 1.57) | |  |  |
| RXRA rs4842196 | A/A | 393 (53.7%) | 774 (54.4%) | Ref. |  | Ref. | | 0.53 | 0.81 |
|  | A/C | 293 (40.0%) | 575 (40.4%) | 1.01 (0.84, 1.21) |  | 0.93 (0.79, 1.09) | |  |  |
|  | C/C | 46 (6.3%) | 75 (5.3%) | 1.23 (0.83, 1.81) |  | 1.16 (0.89, 1.51) | |  |  |
| RXRA rs11103473 | T/T | 319 (43.5%) | 640 (44.8%) | Ref. |  | Ref. | | 0.55 | 0.81 |
|  | A/T | 332 (45.2%) | 635 (44.5%) | 1.04 (0.86, 1.26) |  | 0.97 (0.85, 1.12) | |  |  |
|  | A/A | 83 (11.3%) | 152 (10.7%) | 1.08 (0.80, 1.47) |  | 1.07 (0.88, 1.31) | |  |  |
| RXRA rs1045570 | C/C | 476 (64.9%) | 940 (65.9%) | Ref. |  | Ref. | | 0.58 | 0.81 |
|  | C/A | 234 (31.9%) | 440 (30.8%) | 1.05 (0.86, 1.28) |  | 1.06 (0.86, 1.30) | |  |  |
|  | A/A | 23 (3.1%) | 47 (3.3%) | 0.96 (0.58, 1.60) |  | 0.96 (0.68, 1.36) | |  |  |
| RXRA rs1805343 | A/A | 265 (38.2%) | 515 (39.8%) | Ref. |  | Ref. | | 0.59 | 0.81 |
|  | A/G | 340 (49.1%) | 626 (48.4%) | 1.04 (0.85, 1.27) |  | 0.97 (0.85, 1.12) | |  |  |
|  | G/G | 88 (12.7%) | 153 (11.8%) | 1.10 (0.82, 1.49) |  | 1.07 (0.88, 1.29) | |  |  |
| RXRA rs34622612 | A/A | 606 (82.7%) | 1178 (82.3%) | Ref. |  | Ref. | | 0.60 | 0.82 |
|  | A/G | 121 (16.5%) | 238 (16.6%) | 0.98 (0.78, 1.24) |  | 1.03 (0.72, 1.45) | |  |  |
|  | G/G | 6 (0.8%) | 15 (1.0%) | 0.80 (0.31, 2.06) |  | 0.90 (0.47, 1.71) | |  |  |
| RXRA rs10776909 | G/G | 484 (65.9%) | 969 (67.6%) | Ref. |  | Ref. | | 0.62 | 0.83 |
|  | G/A | 224 (30.5%) | 408 (28.5%) | 1.09 (0.89, 1.32) |  | 1.07 (0.88, 1.30) | |  |  |
|  | A/A | 26 (3.5%) | 57 (4.0%) | 0.92 (0.57, 1.48) |  | 0.95 (0.69, 1.31) | |  |  |
| RXRA rs11185649 | A/A | 416 (56.7%) | 802 (56.1%) | Ref. |  | Ref. | | 0.63 | 0.84 |
|  | G/A | 285 (38.8%) | 551 (38.6%) | 0.98 (0.82, 1.18) |  | 1.03 (0.86, 1.22) | |  |  |
|  | G/G | 33 (4.5%) | 76 (5.3%) | 0.84 (0.56, 1.28) |  | 0.93 (0.70, 1.23) | |  |  |
| RXRA rs6537944 | A/A | 647 (88.8%) | 1257 (88.5%) | Ref. |  | Ref. | | 0.64 | 0.84 |
|  | A/G or G/G | 82 (11.2%) | 164 (11.5%) | 0.98 (0.74, 1.30) |  | 0.97 (0.84, 1.12) | |  |  |
| RXRA rs1805352 | A/A | 350 (47.7%) | 680 (47.4%) | Ref. |  | Ref. | | 0.70 | 0.88 |
|  | A/C | 320 (43.6%) | 623 (43.4%) | 0.99 (0.82, 1.20) |  | 1.00 (0.86, 1.15) | |  |  |
|  | C/C | 64 (8.7%) | 132 (9.2%) | 0.94 (0.69, 1.30) |  | 0.98 (0.79, 1.20) | |  |  |
| RXRA rs3118526 | G/G | 549 (74.8%) | 1050 (73.3%) | Ref. |  | Ref. | | 0.71 | 0.88 |
|  | G/A | 171 (23.3%) | 361 (25.2%) | 0.91 (0.74, 1.12) |  | 0.90 (0.69, 1.17) | |  |  |
|  | A/A | 14 (1.9%) | 22 (1.5%) | 1.18 (0.59, 2.34) |  | 1.14 (0.72, 1.82) | |  |  |
| RXRA rs3118536 | C/C | 502 (68.4%) | 964 (67.4%) | Ref. |  | Ref. | | 0.76 | 0.91 |
|  | C/A | 207 (28.2%) | 421 (29.4%) | 0.94 (0.77, 1.15) |  | 0.93 (0.76, 1.14) | |  |  |
|  | A/A | 25 (3.4%) | 46 (3.2%) | 1.04 (0.64, 1.71) |  | 1.08 (0.77, 1.50) | |  |  |
| RXRA rs3118523 | A/A | 489 (66.7%) | 938 (65.6%) | Ref. |  | Ref. | | 0.85 | 0.96 |
|  | A/G | 219 (29.9%) | 453 (31.7%) | 0.93 (0.77, 1.14) |  | 0.90 (0.73, 1.11) | |  |  |
|  | G/G | 25 (3.4%) | 38 (2.7%) | 1.25 (0.74, 2.10) |  | 1.16 (0.81, 1.66) | |  |  |
| RXRA rs11354233 | D/D | 364 (49.7%) | 721 (50.5%) | Ref. |  | Ref. | | 0.85 | 0.96 |
|  | D/I | 313 (42.7%) | 601 (42.1%) | 1.03 (0.85, 1.24) |  | 1.00 (0.83, 1.21) | |  |  |
|  | I/I | 56 (7.6%) | 105 (7.4%) | 1.05 (0.74, 1.49) |  | 1.05 (0.73, 1.51) | |  |  |
| RXRA rs4240705 | A/A | 298 (40.6%) | 581 (40.5%) | Ref. |  | Ref. | | 0.87 | 0.97 |
|  | A/G | 339 (46.2%) | 675 (47.1%) | 0.98 (0.80, 1.19) |  | 0.95 (0.83, 1.08) | |  |  |
|  | G/G | 97 (13.2%) | 177 (12.4%) | 1.06 (0.80, 1.41) |  | 1.06 (0.89, 1.27) | |  |  |
| RXRA rs3132294 | G/G | 394 (53.7%) | 772 (54.0%) | Ref. |  | Ref. | | 0.89 | 0.97 |
|  | G/A | 295 (40.2%) | 556 (38.9%) | 1.03 (0.86, 1.25) |  | 1.08 (0.92, 1.27) | |  |  |
|  | A/A | 45 (6.1%) | 101 (7.1%) | 0.88 (0.61, 1.26) |  | 0.90 (0.71, 1.15) | |  |  |
| RXRA rs34187476 | G/G | 655 (89.2%) | 1281 (89.4%) | Ref. |  | Ref. | | 0.90 | 0.97 |
|  | C/G or C/C | 79 (10.8%) | 152 (10.6%) | 1.01 (0.75, 1.35) |  | 0.99 (0.85, 1.15) | |  |  |
| RXRA rs12004786 | G/G | 550 (75.3%) | 1066 (75.0%) | Ref. |  | Ref. | | 0.94 | 0.97 |
|  | G/A | 161 (22.1%) | 329 (23.2%) | 0.93 (0.75, 1.16) |  | 0.84 (0.65, 1.07) | |  |  |
|  | A/A | 19 (2.6%) | 26 (1.8%) | 1.45 (0.79, 2.66) |  | 1.30 (0.86, 1.98) | |  |  |
| RXRA rs1007971 | G/G | 454 (61.9%) | 881 (61.6%) | Ref. |  | Ref. | | 0.96 | 0.97 |
|  | C/G | 251 (34.2%) | 503 (35.2%) | 0.98 (0.81, 1.18) |  | 0.93 (0.76, 1.14) | |  |  |
|  | C/C | 28 (3.8%) | 46 (3.2%) | 1.19 (0.73, 1.94) |  | 1.11 (0.79, 1.55) | |  |  |
| RXRA rs34857233 | G/G | 639 (87.2%) | 1246 (87.0%) | Ref. |  | Ref. | | 0.95 | 0.97 |
|  | G/A | 90 (12.3%) | 178 (12.4%) | 0.98 (0.75, 1.28) |  | 1.00 (0.64, 1.55) | |  |  |
|  | A/A | 4 (0.5%) | 8 (0.6%) | 1.00 (0.30, 3.31) |  | 1.00 (0.44, 2.27) | |  |  |
| RXRA rs11102986 | G/G | 517 (70.5%) | 1017 (71.0%) | Ref. |  | Ref. | | 1.00 | 1.00 |
|  | A/G | 196 (26.7%) | 372 (26.0%) | 1.03 (0.84, 1.26) |  | 1.03 (0.83, 1.27) | |  |  |
|  | A/A | 20 (2.7%) | 44 (3.1%) | 0.90 (0.53, 1.54) |  | 0.96 (0.67, 1.38) | |  |  |
| CYP27B1 rs4646536 | A/A | 303 (41.3%) | 573 (40.0%) | Ref. |  | Ref. | | 0.91 | 0.97 |
|  | A/G | 332 (45.3%) | 663 (46.3%) | 0.95 (0.78, 1.14) |  | 1.01 (0.89, 1.15) | |  |  |
|  | G/G | 98 (13.4%) | 196 (13.7%) | 0.93 (0.71, 1.24) |  | 0.98 (0.82, 1.18) | |  |  |

a. Conditional logistic regression, matching factors: age and menopausal status at blood donation (partial matching)

b. Conditional logistic regression, matching factors: age and menopausal status at blood donation (partial matching), covariates: age at menarche (continuous), family history of breast cancer (yes/no), BMI (log transformed), hormone replacement therapy (HRT, ever/never), and full term pregnancy (ordered, age at first full term pregnancy ≤20 years, 21-25 years, 26-30 years, > 30 years, nulliparous). Missing data for the following covariates was imputed using fully conditional specification multiple imputation: age at menarche, BMI, HRT, and age at first full term pregnancy.

c. P values for trend were calculated with 0, 1, 2 (or 0, 1) corresponding to the genotypes and were two-sided

d. False discovery rate, calculated by using the linear step-up method of Benjamini and Hochberg in SAS (PROC MULTTEST)
