# Supplementary material for: Genetic Polymorphisms in Vitamin D Metabolism and Signaling Genes and Risk of Breast Cancer: A Nested Case-Control Study
Source: PLoS One. 2015 Oct 21;10(10):e0140478. doi: 10.1371/journal.pone.0140478 (PMC4619526; doi:10.1371/journal.pone.0140478)
Supplement: S3 Table — (DOCX) [file pone.0140478.s004.docx]

S3 Table. Haplotypes of VDR, RXRA, and CYP24A1 and risk of breast cancer

| Gene block  Haplotype | Estimated haplotype frequency (%) | | Age- and menopausal status-adjusted ^a^ | Multivariate-adjusted ^b^ | | | | | | |  |
| --- | --- | --- | --- | --- | --- | --- | --- | --- | --- | --- | --- |
|  | Cases | Controls | OR (95%CI) | OR (95%CI) | | P value ^c^ | | FDR^f^ | | |  |
| **VDR** |  |  |  |  | |  | |  | | |  |
| Block 1 (rs9729, rs3847987, rs757343, rs1544410 [Bsm1], rs11574085, rs11168267, rs11574077) | | | | | | | | | | |  |
| CCGGAGA | 36.7% | 39.3% | Reference | Reference | | |  | | |  | |
| ACGAAGA | 35.8% | 34.6% | 1.11 (0.96, 1.29) | 1.12 (0.96, 1.30) | | | 0.16 | | | 0.54 | |
| AAAGAAA | 13.0% | 11.2% | 1.25 (1.02, 1.55) | 1.27 (1.03, 1.57) | | | 0.03 | | | 0.42 | |
| ACGAAGG | 5.8% | 5.7% | 1.12 (0.85, 1.50) | 1.16 (0.87, 1.55) | | | 0.31 | | | 0.69 | |
| CCGGTGA | 5.0% | 5.5% | 0.98 (0.73, 1.32) | 0.99 (0.73, 1.33) | | | 0.94 | | | 0.94 | |
| AAAGAGA | 2.1% | 2.1% | 1.03 (0.66, 1.61) | 1.03 (0.65, 1.62) | | | 0.91 | | | 0.94 | |
| Rare ^e^ |  |  | 1.24 (0.71, 2.17) | 1.28 (0.73, 2.26) | | | 0.39 | | | 0.74 | |
| Block 2 (rs2239182, rs2107301, rs2283342, rs2239180) | | | | | | | | | | |  |
| GGAG | 44.2% | 43.1% | Reference | Reference | | |  | |  | | |
| AGAG | 17.7% | 17.6% | 0.96 (0.80, 1.16) | 0.95 (0.78, 1.14) | | | 0.56 | | 0.76 | | |
| AAAG | 11.0% | 12.8% | 0.84 (0.68, 1.05) | 0.85 (0.68, 1.05) | | | 0.14 | | 0.53 | | |
| AAGG | 10.1% | 10.4% | 0.95 (0.76, 1.18) | 0.92 (0.74, 1.15) | | | 0.47 | | 0.76 | | |
| AAAC | 10.3% | 8.4% | 1.17 (0.92, 1.47) | 1.18 (0.93, 1.49) | | | 0.17 | | 0.54 | | |
| AGGG | 1.8% | 2.1% | 0.94 (0.56, 1.57) | 0.93 (0.56, 1.56) | | | 0.79 | | 0.91 | | |
| Rare ^e^ |  |  | 0.85 (0.61, 1.19) | 0.84 (0.60, 1.17) | | | 0.30 | | 0.69 | | |
| Block 3 (rs2238136, rs2853564, rs11574032) | | | | | | | | | | |  |
| GGG | 42.1% | 42.4% | Reference | Reference |  | | |  | | |  |
| GAG | 29.9% | 31.9% | 0.95 (0.81, 1.10) | 0.95 (0.82, 1.11) | | | 0.54 | | 0.76 | | |
| AAG | 20.0% | 18.7% | 1.07 (0.91, 1.27) | 1.08 (0.91, 1.29) | | | 0.37 | | 0.74 | | |
| AAA | 8.0% | 7.0% | 1.14 (0.89, 1.46) | 1.12 (0.87, 1.43) | | | 0.39 | | 0.74 | | |
| Block 4 (rs11574026, rs3890733, rs10875695, rs4760655) | | | | | | | | | | |  |
| GACA | 37.2% | 38.2% | Reference | Reference | | |  | |  | | |
| GGCG | 36.4% | 33.0% | 1.13 (0.98, 1.31) | 1.13 (0.97, 1.31) | | | 0.12 | | 0.50 | | |
| GGAA | 15.9% | 17.7% | 0.94 (0.78, 1.12) | 0.93 (0.78, 1.12) | | | 0.45 | | 0.76 | | |
| AGAA | 7.5% | 7.2% | 1.07 (0.83, 1.38) | 1.11 (0.86, 1.43) | | | 0.44 | | 0.76 | | |
| GGCA | 3.0% | 3.9% | 0.81 (0.56, 1.17) | 0.79 (0.55, 1.15) | | | 0.22 | | 0.62 | | |
| Block 5 (rs7299460, rs7139166, rs11568820 [Cdx2]) | | | | | | | | | | |  |
| GGG | 38.2% | 38.8% | Reference | Reference | | |  | |  | | |
| GCG | 35.3% | 32.2% | 1.11 (0.96, 1.30) | 1.11 (0.95, 1.30) | | | 0.18 | | 0.54 | | |
| ACA | 17.3% | 18.4% | 0.97 (0.81, 1.16) | 0.98 (0.81, 1.17) | | | 0.80 | | 0.91 | | |
| AGG | 7.5% | 7.8% | 0.98 (0.75, 1.28) | 0.99 (0.75, 1.30) | | | 0.92 | | 0.94 | | |
| ACG | 1.7% | 2.7% | 0.68 (0.39, 1.18) | 0.63 (0.35, 1.11) | | | 0.11 | | 0.50 | | |
| **RXRA** |  |  |  |  | | |  | |  | | |
| Block 1 (rs4917348, rs11185649, rs12339187, rs12006409) | | | | | | | | | | |  |
| AAAG | 76.1% | 75.3% | Reference | Reference | | |  | |  | | |
| AGGA | 5.9% | 7.9% | 0.73 (0.57, 0.95) | 0.74 (0.57, 0.96) | | | 0.02 | | 0.42 | | |
| AGAG | 5.3% | 5.0% | 1.07 (0.79, 1.44) | 1.10 (0.81, 1.49) | | | 0.55 | | 0.76 | | |
| GGGG | 4.6% | 3.9% | 1.18 (0.86, 1.62) | 1.19 (0.87, 1.65) | | | 0.28 | | 0.69 | | |
| GGAG | 4.3% | 3.9% | 1.09 (0.80, 1.49) | 1.11 (0.80, 1.52) | | | 0.53 | | 0.76 | | |
| AGAA | 3.3% | 3.8% | 0.89 (0.62, 1.26) | 0.90 (0.63, 1.29) | | | 0.56 | | 0.76 | | |
| Block 2 (rs11102986, rs11103473, rs10776909, rs12004589) | | | | | | | | | | |  |
| GTGC | 66.1% | 67.0% | Reference | Reference | | |  | |  | | |
| GAAC | 2.7% | 2.2% | 1.22 (0.82, 1.82) | 1.23 (0.82, 1.85) | | | 0.31 | | 0.69 | | |
| GAGA | 9.7% | 10.5% | 0.93 (0.75, 1.15) | 0.93 (0.75, 1.16) | | | 0.53 | | 0.76 | | |
| GAGC | 5.5% | 4.3% | 1.29 (0.97, 1.73) | 1.27 (0.94, 1.70) | | | 0.12 | | 0.50 | | |
| AAAC | 16.1% | 16.0% | 1.02 (0.86, 1.22) | 1.03 (0.86, 1.23) | | | 0.73 | | 0.90 | | |
| Block 3 (rs3118536, rs12004786, rs4240705) | | | | | | | | | | |  |
| CGA | 63.1% | 63.3% | Reference | Reference | | |  | |  | | |
| AGG | 17.3% | 17.7% | 0.98 (0.83, 1.16) | 0.98 (0.83, 1.17) | | | 0.86 | | 0.93 | | |
| CAG | 13.4% | 12.9% | 1.04 (0.86, 1.26) | 1.03 (0.85, 1.25) | | | 0.76 | | 0.91 | | |
| CGG | 5.6% | 5.3% | 1.06 (0.79, 1.41) | 1.03 (0.77, 1.38) | | | 0.83 | | 0.92 | | |
| **CYP24A1** |  |  |  |  | | |  | |  | | |
| Block 1 (rs2762934, rs927650, rs6097809, rs1570669, rs2296239) | | | | | | | | | | |  |
| GAAAG | 49.5% | 46.1% | Reference | Reference | | |  | |  | | |
| AGAAG | 16.7% | 18.6% | 0.83 (0.70, 0.99) | 0.82 (0.68, 0.98) | | | 0.03 | | 0.42 | | |
| GGAGA | 15.0% | 16.8% | 0.83 (0.69, 1.00) | 0.84 (0.70, 1.01) | | | 0.07 | | 0.42 | | |
| GGAGG | 12.1% | 12.0% | 0.94 (0.77, 1.14) | 0.95 (0.78, 1.17) | | | 0.64 | | 0.84 | | |
| GGGGA | 6.6% | 6.4% | 0.96 (0.74, 1.24) | 0.95 (0.73, 1.25) | | | 0.73 | | 0.90 | | |
| Block 2 (rs4809960, rs2296241, rs2245153) | | | | | | | | | | |  |
| AGA | 50.4% | 46.5% | Reference | Reference | | |  | |  | | |
| AAA | 29.8% | 30.9% | 0.89 (0.77, 1.03) | 0.87 (0.75, 1.01) | | | 0.07 | | 0.42 | | |
| GAG | 15.3% | 16.8% | 0.84 (0.70, 1.01) | 0.83 (0.69, 1.00) | | | 0.05 | | 0.42 | | |
| GAA | 4.5% | 5.8% | 0.72 (0.53, 0.97) | 0.73 (0.54, 0.99) | | | 0.04 | | 0.42 | | |

a. Unconditional logistic regression adjusted for age (continuous) and menopausal status (pre-/peri- vs. post-menopausal) at blood donation

b. Unconditional logistic regression adjusted for age (continuous), menopausal status (pre-/peri- vs. post-menopausal) at blood donation, covariates: age at menarche (continuous), family history of breast cancer (yes/no), BMI (log transformed), hormone replacement therapy (HRT) use (ever/never), and full term pregnancy (ordered, age at first full term pregnancy ≤ 20 years, 21-25 years, 26-30 years, > 30 years, nulliparous). Missing data for the following covariates was imputed using fully conditional specification multiple imputation: age at menarche, BMI, HRT, and age at first full term pregnancy.

c. False discovery rate calculated by using the linear step-up method of Benjamini and Hochberg in SAS (PROC MULTTEST)

d. Haplotypes with estimated frequency ≥ 2% were included as single haplotype in analyses;   rare haplotypes with frequencies < 2% were pooled in rare category and included in the analysis if the sum of the rare frequencies was ≥ 1%
